# Communication approaches to enhance patient motivation and adherence in cardiovascular disease prevention

**DOI:** 10.1002/clc.23555

**Published:** 2021-08-20

**Authors:** Eamon Y. Duffy, Dominique Ashen, Roger S. Blumenthal, Dorothy M. Davis, Martha Gulati, Michael J. Blaha, Erin D. Michos, Khurram Nasir, Miguel Cainzos‐Achirica

**Affiliations:** ^1^ Department of Internal Medicine Johns Hopkins University School of Medicine Baltimore Maryland USA; ^2^ Johns Hopkins Ciccarone Center for the Prevention of Cardiovascular Disease, Division of Cardiology Johns Hopkins University School of Medicine Baltimore Maryland USA; ^3^ School of Nursing Johns Hopkins University Baltimore Maryland USA; ^4^ University of Arizona College of Medicine Phoenix Arizona USA; ^5^ Banner University Medical Center Phoenix Arizona USA; ^6^ Welch Center for Prevention, Epidemiology and Clinical Research Johns Hopkins University Baltimore Maryland USA; ^7^ Department of Epidemiology Johns Hopkins Bloomberg School of Public Health, Johns Hopkins University Baltimore Maryland USA; ^8^ Division of Cardiovascular Prevention and Wellness, Department of Cardiology Houston Methodist DeBakey Heart & Vascular Center Houston Texas USA; ^9^ Center for Outcomes Research Houston Methodist Houston Texas USA

**Keywords:** advocacy, behavioral science, cardiovascular disease, lifestyle, prevention, psychology

## Abstract

Preventive cardiology visits have traditionally focused on educating patients about disease risk factors and the need to avoid and manage them through lifestyle changes and medications. However, long‐term patient adherence to the recommended interventions remains a key unmet need. In this review we discuss the rationale and potential benefits of a paradigm shift in the clinician‐patient encounter, from focusing on education to explicitly discussing key drivers of individual motivation. This includes the emotional, psychological, and economic mindset that patients bring to their health decisions. Five communication approaches are proposed that progress clinician‐patient preventive cardiology conversations, from provision of information to addressing values and priorities such as common health concerns, love for the family, desire of social recognition, financial stressors, and desire to receive personalized advice. Although further research is needed, these approaches may facilitate developing deeper, more effective bonds with patients, enhance adherence to recommendations and ultimately, improve cardiovascular outcomes.

**Abbreviations**
ACS: acute coronary syndrome
ASCVD: atherosclerotic cardiovascular disease
CVD: cardiovascular disease

## 1 INTRODUCTION

Compelling science has demonstrated the cardiovascular benefits of regular physical activity and healthy food choices.^1^, ^2^, ^3^, ^4^ Also, a myriad of pharmacotherapies are now available which dramatically decrease the incidence of cardiovascular disease (CVD) events in symptomatic patients, as well as among asymptomatic apparently healthy persons at high risk.^5^, ^6^, ^7^, ^8^ However, sustained healthy lifestyle change and long‐term adherence to cardiovascular medications remain key unmet needs in preventive cardiology,^9^, ^10^ limiting the effectiveness of these otherwise powerful interventions.

Socioeconomic factors are key determinants of the incidence of CVD at the population level.^11^ Also, culture strongly influences the behaviors of individuals of a given society, with direct implications for daily habits such as dietary choices and levels of physical activity. Consequently, preventive interventions at the policy and population level can have large benefits.^11^ Still, marked heterogeneity in lifestyle choices exists among persons of a given country, culture, religion, and socioeconomic status.^12^ Thus, identification of the determinants which drive individual‐level behavioral choices becomes crucial to inform effective personalized prevention.

**FIGURE 1:**
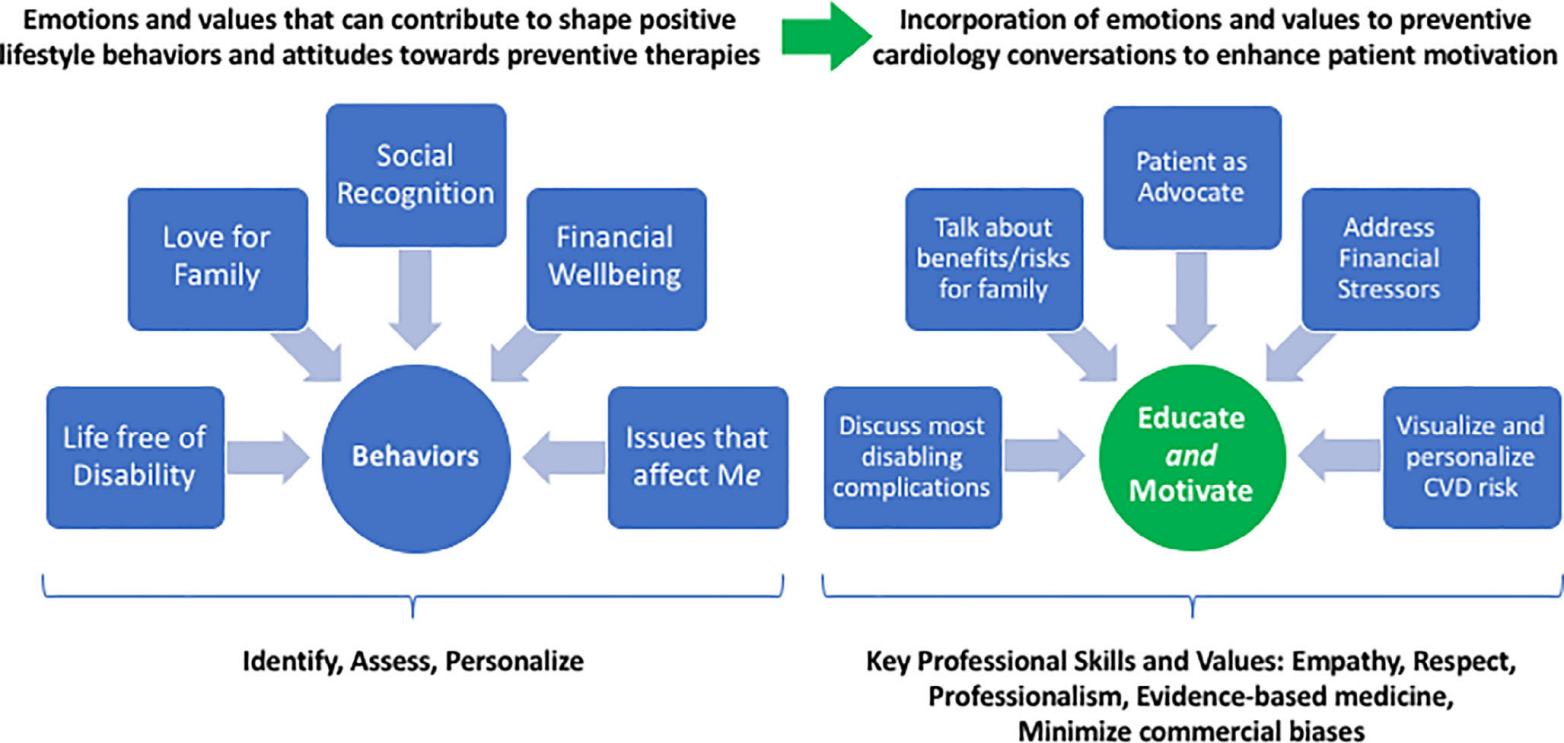
Central Illustration. Patient behaviors are formed by a variety of values, priorities, and stressors. Through targeted conversation, clinicians can identify these drivers of behavior and go beyond education to trigger motivation. Abbreviation: CVD, cardiovascular disease

Emotions and personal values represent key drivers of individual human behavior (Figure 1).^13^ These shape our priorities and motivations, and inform many of our decisions, including those which lead persons to behave differently from what is typical of their culture, or accepted as the established social or group norm. Because of their power, these triggers are pursued by marketing specialists and are targeted in commercial advertising. The same may be true for personalized communication approaches, which are also used as a powerful commercial marketing strategy.^14^ For similar reasons, targeting these triggers may represent a powerful opportunity to shape long‐term habits and increase adherence to medications among cardiovascular patients.

In this review we describe key human emotions and values relevant to daily behaviors, many of which have an impact on cardiovascular health and can be serve as the foundation for motivational prevention discussions with patients. Building on those, we discuss opportunities to engage in more personalized, tailored, meaningful conversations about CVD health with patients, aimed at further enhancing their motivation to adhere to recommended risk management interventions and eventually, improve outcomes.

## 2 A PARADIGM SHIFT FROM EDUCATION TO MOTIVATION

The Framingham Heart Study was the first to establish the importance of hypertension, diabetes and other risk factors as causes of CVD.^15^ In subsequent decades, the medical community's understanding of the importance of these health hazards only grew deeper, whereas knowledge about the importance of daily lifestyle habits in mitigating these CVD risk factors remained limited among the general public. This information gap led to a clinician‐patient communication paradigm under which clinicians delivering preventive cardiology care would use most of the encounter to *educate* their patients about the causes, mechanisms and consequences of CVD—and on the need to avoid them.

Although this communication paradigm still prevails, the public has caught up. Most people now have free, continuous access to multiple health information resources, from television and other mass media to online search engines, electronic patient portals, and social media. More than half of Americans report hearing news about how to prevent serious diseases on at least a weekly basis, with elderly patients reporting even greater awareness.^16^ Notions such as the cardiovascular health risks of saturated fats, tobacco use, or insufficient physical activity have now become part of our culture, and health conversations are ubiquitous. When considering a medical treatment, the majority of Americans now do their own research on the topic in addition to consulting with a doctor.^17^ More than 95% of Americans, when polled, believe that healthy eating habits and sufficient exercise are important in preventing serious diseases such as cancer or heart disease.^17^ Although significant work remains to be done in this area, we would argue that our patients have never been better informed or had access to more information about their health.

It is important to note, however, that studies have found that with the increased availability of medical information has come the increased availability of medical misinformation.^18^ Therefore, patient education should remain a top priority. Specifically, providers should pay special attention to identify and address health beliefs not supported by high‐quality data, as those may impair patients' motivation to adhere to healthier lifestyles and to effective, guideline‐recommended medications such as statins.

**FIGURE 2:**
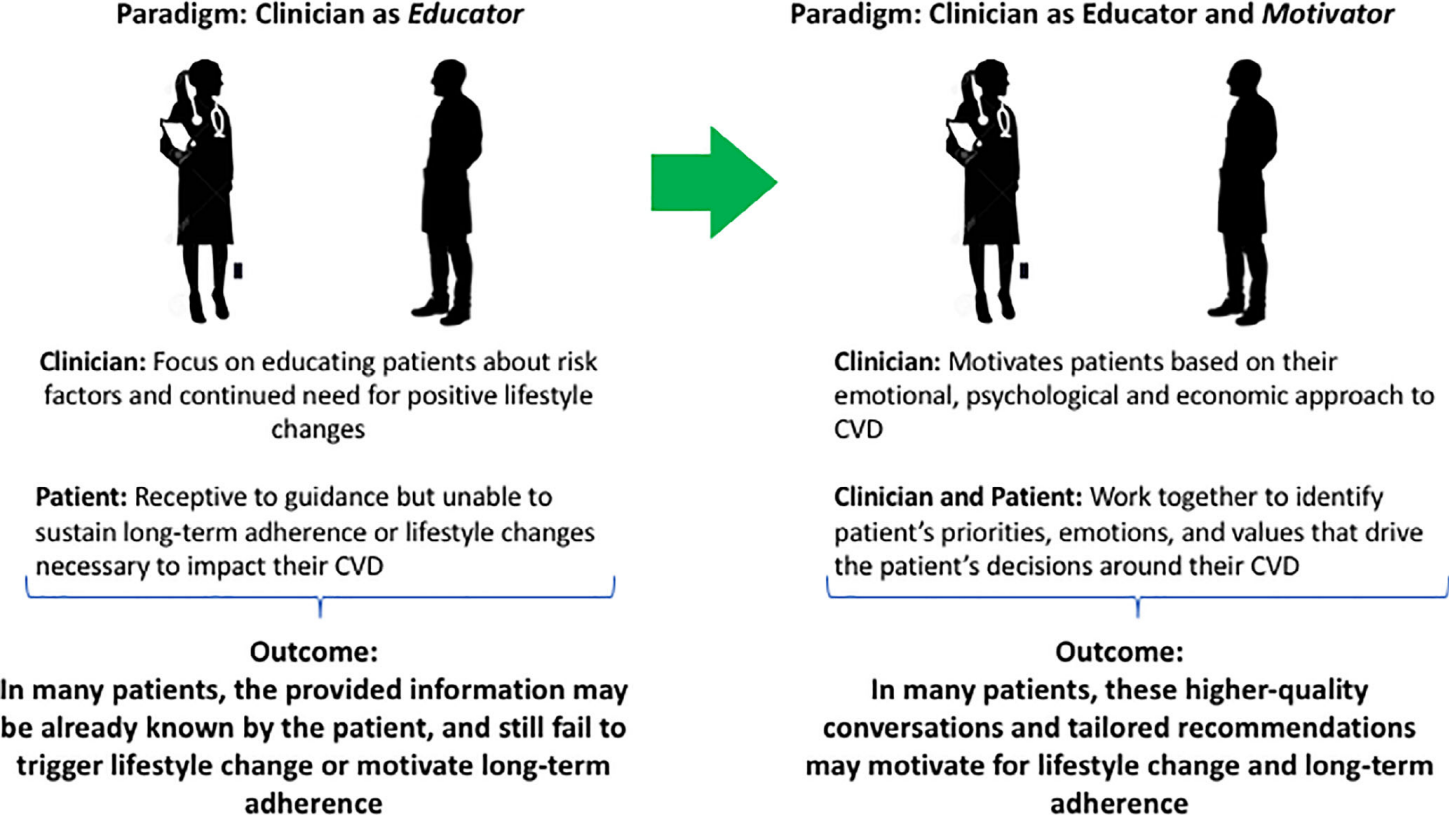
A paradigm shift in preventive cardiology discussions: from education to motivation. The traditional focus on educating the patient about cardiovascular risk factors can expand to include pinpointing the values, emotions, and stressors that motivate how that individual makes decisions about their cardiovascular health. Abbreviation: CVD, cardiovascular disease

This evolution of awareness calls for an evolution in how preventive cardiology professionals engage with their patients about their health. Of note, despite this wave of insight, the prevalence of key metabolic risk factors such as sedentarism, obesity, or diabetes continue on the rise.^19^ The effectiveness of long‐term tobacco cessation interventions remains low even among patients who have had a myocardial infarction, as is adherence to other lifestyle recommendations and life‐saving medications.^9^, ^10^ Clinicians themselves, who have the highest level of education on health, do only slightly better in adherence to medical recommendations than do non‐clinicians.^16^ These unsatisfying trends suggest that the traditional “clinician as educator” paradigm, although crucial, may fall short in attaining fully effective, sustained change in many patients. It may thus be the time to expand the role of preventive cardiovascular professionals from *educators* to *motivators*, able to further connect with the patient's needs, priorities and values in an even more meaningful, powerful manner (Figure 2).

Under this novel approach, professionals would be trained and empowered to connect the insidious diseases that we aim to prevent with the aspects of our patient's lives that are most important to them, shifting the focus from what patients *know* to what they actually *care about*. Emotions are not extraneous to preventive cardiology professionals—for instance, patient consultations are often triggered by the concern caused by a recent event affecting a patient's close friend or relative. Also, a focus on motivation is already used by some specialized physicians and nurses in some prevention clinics. Motivational interviewing, introduced by William Miller in 1983 to engage with people with alcohol use disorder, is a common practice in the substance use field but has yet to reach its full potential in the field of CVD prevention.^20^ This approach is “a collaborative conversation style for strengthening a person's own motivation and commitment to change.”^20^ Studies have shown significant improvements in substance use outcomes and in increasing physical activity,^21^ but a recent meta‐analysis of motivational interviewing to support risk factor modification in patients at increased risk for CVD concluded that the effectiveness of this approach in this population remains uncertain and more robust studies are required.^22^ It is important to note, however, that the approaches discussed in this review were not formally assessed in any of those studies.

Additionally, most clinicians lack training and experience on how to engage in these conversations. To ameliorate this, below we present five communication approaches that may be considered to further enhance preventive cardiology discussions with patients (Table 1). More than a call to action, these approaches can serve as concrete topics that clinicians can discuss with patients to motivate lasting change.

**TABLE 1:** Summary of proposed communication opportunities aimed at increasing the patient's motivation to implement healthy lifestyle changes and adhere to recommended preventive medications

Communication opportunities
“It's not *only* about CVD”—Discuss additional downstream conditions such as dementia, stroke, heart failure, erectile dysfunction associated with CVD risk factors
“Your choices directly impact those you care about”—Incorporating the health of loved ones to the conversation
“We want you!”—Empower appropriate patients to serve as CVD advocates and champions within their social networks
“Let us talk about money”—Address the impact of CVD on financial security, a primary stress in our patients' lives
“Let us talk about *you*”—Further personalize the conversation to the specific patient, for example, discuss sex‐specific risk factors/risk enhancers or burden of subclinical CVD

Abbreviation: CVD, cardiovascular disease.

### 2.1 #1: Expanding the patient's awareness about the effects of cardiovascular risk factors on other conditions—“It's not *only* about CVD”

The importance of tobacco, diabetes, sedentarism or unhealthy diets as key risk factors for CVD have been widely publicized. Despite this, the indolent, mostly asymptomatic nature of these chronic processes can make them easily overlooked or discounted by patients, who often underestimate their risk.^23^, ^24^ Even when a myocardial infarction or a stroke eventually occur, both of which can be life‐changing events,^25^ large clinical registries such as EUROASPIRE V suggest that long‐term adherence to recommended changes is frustratingly low.^9^

In the last few years, research has identified the detrimental effects that traditional cardiovascular risk factors also have as causes of other conditions, such as dementia,^26^ vascular erectile dysfunction,^27^ several types of cancer,^28^, ^29^ and loss of quality of life, among others (Table 2). Importantly, according to recent studies, these and their disabling effects represent greater health concerns to patients than CVD itself.^30^ This may be due to the fact that patients consistently misunderstand their long CVD risk, with studies showing 40% of patients underestimate their risk.^31^ This underestimation leads to undermotivation to adhere to primary and secondary CVD prevention efforts.

**TABLE 2:** Non‐CVD associations between traditional cardiovascular risk factors and non‐cardiovascular conditions

Non‐CVD consequences of cardiovascular risk factors
Cancer
Pregnancy complications
Erectile and other sexual dysfunction
Chronic kidney injury, need for hemodialysis
Obstructive pulmonary disease
Cognitive decline
Dementia
Depression
Neuropathy
Cataracts, glaucoma
Loss of quality of life
Reduced mobility
Disability
Death

Abbreviation: CVD, cardiovascular disease.

Although during preventive cardiology clinician‐patient discussions most professionals intuitively tend to focus on informing about CVD as the key condition to be avoided, incorporation of these other, highly relevant outcomes to routine discussions may help increase the patient's motivation to change. Appropriate personalization of the information being provided, focusing on the most relevant outcomes to each patient could be used to enhance motivation even further. For example, provision of information about erectile dysfunction to young male smoker or impaired workplace performance to working‐aged patients may be an effective trigger of meaningful change for a patient. If preventing CVD does not motivate a patient, then the possibility to also reduce the risk of dementia or erectile dysfunction or disability impacting work productivity through healthy lifestyle habits may help do so.

In addition, scientific societies and health agencies should consider stressing, as part of their health communication initiatives, the notion of traditional “cardiovascular” risk factors also being risk factors of multiple, disabling, and distressing diseases. This may be a powerful approach to increase awareness and motivation for healthy lifestyle change in the large, general population.

### 2.2 #2: Incorporating the health of the loved ones to the conversation—“Your choices directly impact those you care about”

Patients may perceive their lifestyle choices as individual, personal decisions. However, a person's lifestyle has direct implications for the health of their relatives and social network. Examples abound: secondhand smoking is a strong, independent risk factor of CVD, cancer, and other conditions,^32^ eating patterns often cluster in families,^33^ and so do attitudes toward physical activity.^33^, ^34^

Love and care for other beings is one of the strongest human emotions.^17^

Incorporation of this key driver to personal health decision‐making can therefore have very powerful effects. Paradigmatic examples of this are the lifestyle changes often observed among pregnant women, who will often quit tobacco and alcohol use immediately after learning they are expecting a baby.^35^ The patient's family has indeed become an active area of research in preventive cardiology in recent years.^36^ For instance, studies have detailed the immense burden that CVD places on informal caregivers, also known as family members. A recent review on the topic reported that such care led to a compromised career (>50%), inability to work (48%), deprioritization of work (33%), and negative impact on their disposable income (>94%).^37^

Clinicians may therefore want to carefully but explicitly discuss with their patients the detrimental consequences that specific habits may have not *only* for them, but also for the patient's family and network. Multiple, relevant notions should be discussed, from the harm that smoking tobacco products cause on the people around them, to the consequences of educating, by example, children on eating unhealthy foods. The opposite should also be discussed, that is, the potential positive familial and social consequences of implementing individual healthy lifestyle changes. Building on this information, love for those persons could be incorporated to the conversation as a very powerful motivator and trigger for healthy change.

In addition, clinicians may also want to stress the impact that conditions such as CVD, cancer, or dementia may have in terms of reducing the time that the patient might be able to spend with their partner, children, grandchildren, and other beloved ones. CVD risk factors markedly shorten life expectancy,^38^ and emphasizing the opportunity cost that the eventual development of established CVD and other conditions represents in terms of “*years of joy lost*” in a patient's life may also be a powerful strategy to further stimulate healthy changes.

### 2.3 #3: “We want you!”—for CVD prevention

A person's engagement in a given activity increases when they are assigned an active, leadership role.^39^ Social recognition for positive behaviors is a powerful determinant of human behavior since childhood.^40^ In several European countries, “expert patients” with diabetes who teach their peers to use insulin pumps usually have optimal glucose control, and are a powerful aid for endocrinologists to help enhance the use of medications by other patients.^41^ Building on these notions, as well as on some of the approaches described in the above sections, another innovative communication opportunity for enhanced CVD prevention may be to engage select patients attending preventive cardiology clinics as cardiovascular health experts and champions within their families and communities. Examples of ongoing initiatives include “Women Heart Champions” which are women with CVD who are trained to be spokeswomen in their communities and hospitals, and the initiatives promoted by the Familial Hypercholesterolemia Foundation. Similar to how patients with substance use disorder are often offered a peer recovery coach, the period following an acute cardiac event or stroke could be a window of opportunity to connect patients with their peer CVD coach. These connections could be made in person or virtually with online platforms or even social media. To avoid damaging misinformation, discussed previously, these connections may need to be supervised by health professionals to ensure exchange of evidence‐based information.

**FIGURE 3:**
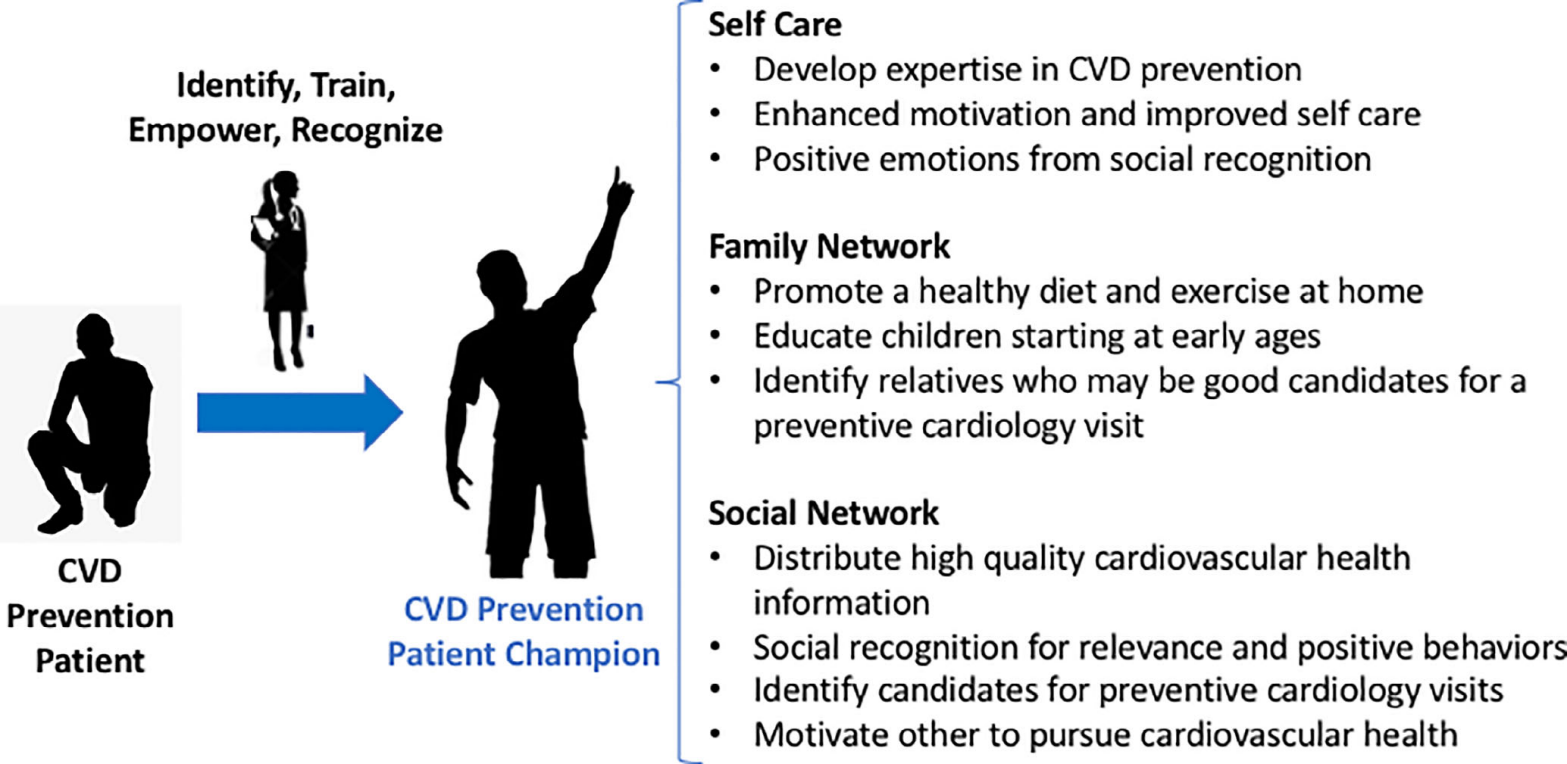
Engaging the patient as a healthy lifestyle advocate and champion. Patient advocates become more active patients and positively impact their family and social networks, creating a ripple effect of cardiovascular prevention. Abbreviation: CVD, cardiovascular disease

This approach has three key potential benefits. First, it would likely increase the patient's engagement and motivation to adhere to a healthy lifestyle even further. Second, it could multiply the impact of the provided recommendations, as they would reach not only the patient attending the clinic but also other members of their family and social network, many of whom will also benefit from those recommendations and whom would not have been reached otherwise (Figure 3). Third, it creates peer‐group based care with CVD patients engaging with one another and empathizing with barriers to progress. A recent randomized trial by Fuster and colleagues found that such peer‐group interventions had beneficial effects on CVD risk factors.^42^ Additionally, a recent study of an “Expert Patient Program” on post‐MI patients over 2 years found low participation but significant improvement in the quality of life in the intervention group.^43^

Importantly, selection of such patient advocates would have to be done carefully. Despite expanded health knowledge, some misconceptions prevail among the general public with regards, for example, to the benefits of specific foods and diets. Therefore, optimal candidates would have to be provided with training and followed with frequent meetings and refreshers. This would be particularly important for patients who may use social media to maximize the reach of their advocacy. Nonetheless, with appropriate selection, training and supervision, this approach could have enormous, multiplicative benefits.

Preventive cardiology patients should be particularly encouraged to discuss CVD prevention insights with their children and younger relatives. Young patients now account for an increasing proportion of myocardial infarction hospitalizations, and middle‐aged women have seen the fastest relative increase in heart attacks over recent years.^44^ To truly curb the impact of CVD it is crucial to prevent its development in individuals in their 30s and 40s as aggressively as we treat it's comorbidities later in life. However, the challenge is that these younger patients often do not present to preventive cardiology clinics. Health professionals are in a strong position to not only guide the patient in front of them, but also become the cardiologist to that person's entire family. This may have a preventive ripple effect throughout families and society, amplifying guidance, motivation, and knowledge so older patients who *want* to hear it and younger non‐patients who *need* to hear it improve their CVD health the same.

### 2.4 #4: “Let's talk about money”

Finances are one of the main concerns of Americans, which makes it a strong determinant of individual decision‐making.^45^ Health is the number one cause of financial hardship in the US,^46^ and CVD and its risk factors are major causes of “financial toxicity.”^47^, ^48^ Even non‐elderly patients with health insurance face high financial distress due to CVD‐related medical costs. For example, a hospitalization for an acute myocardial infarction requiring percutaneous coronary intervention costs in average 20 000 US dollars,^49^ and most novel cardiovascular medications are expensive and prescribed indefinitely. Many of the same interventions that help to prevent ASCVD also help to prevent heart failure, a growing source of financial toxicity in this country.^50^ Recent work has estimated the out of pocket cost of angiotensin receptor‐neprilysin inhibitor therapy at $1600. As the coveted guideline directed medical therapy becomes ever more expensive, it is no wonder one in eight adults with atherosclerotic CVD report cost‐related medication non‐adherence.^51^

In this context, clinician‐patient encounters could be framed to include careful, evidence‐based, data‐driven discussions on the financial implications that poor management of risk factors and CVD events can have for the patient and their family. Using a constructive approach, the discussion could focus on the benefits, in terms of savings, of avoiding hospitalizations, costly medical procedures, and need for lifelong pharmacotherapies. The large potential savings brought by healthy changes such as quitting tobacco could also be discussed with active smokers. All of these notions may have powerful effects shaping healthy behaviors.

It could be argued that provision of this kind of information, which is widely available in the medical and financial literature, should be part of standard counseling in preventive cardiology visits. However, barriers to its provision are multiple, including limited visit time and lack of specific training among some professionals. Discussing financial security is a delicate topic, one that most clinicians and patients may be willing to avoid. Training should be provided to ensure that these conversations happen in the most empathetic, respectful, evidence‐based manner. In doing so we aim to motivate our patients by addressing one of their primary life stressors head on. If cost of care is driving non‐adherence, and studies show clearly that it is, then clinicians should be empowered to discuss this central topic to the lives, and health, of our patients.

### 2.5 #5: Personalizing communication even further—“Let's talk about you”

Additional innovative approaches may be used to further personalize risk communication, aimed at maximizing its relevance to the individual patient seeking preventive cardiology care. Men and women are impacted by CVD risk factors in different ways, and some, such as smoking and diabetes, confer a greater relative risk for CVD in women than they do in men.^52^ Also, certain risk enhancers such as preterm delivery, gestational diabetes, and pre‐eclampsia are unique to women. Furthermore, CVD risk rises sharply in women after menopause, creating a unique opportunity to address CVD risk at a key turning point in a woman's life.^53^ Acknowledging sex‐specific differences in the risk of CVD and introducing values that may be particularly relevant to women as part of clinician‐patient discussions with female patients may result in a more meaningful, effective communication. In addition to tailoring such conversations by gender, these conversations can be adapted toc hanging priorities with age and cognitive function of the patient, recognizing that goals of care change as patients age.

Evolving medical technologies can also play a powerful role improving patients' motivation for lifestyle change or for adhering to preventive pharmacotherapies. Visualization of subclinical atherosclerotic plaque, which is strongly associated with increased risk of CVD events, using tools such as computed tomography for coronary artery calcium scoring has demonstrated to improve patient motivation and adherence.^54^ Consistent with this, current clinical practice guidelines from the American College of Cardiology/American Heart Association acknowledge that patients reluctant to take a statin might benefit from coronary calcium scoring, making a stronger case for therapy should established disease be demonstrated.^10^

Beyond the communication that takes place at the prevention clinic, which is usually brief and happens sporadically, mobile health, and wearable technologies provide an opportunity to sustain communication and therefore motivation on a daily basis, particularly if a health messaging component is included.^55^ In a recent randomized trial, an automated tracking–texting intervention that provided participants with tailored health messaging and physical activity recommendations, patients increased the average levels of physical activity as compared to tracking alone.^56^ Used wisely, these technologies provide a tremendous opportunity to strengthen patients' motivation between onsite (or online) medical visits. Additionally, as smartphones and screen time continue to absorb more and more of our patients' time and attention,^57^ these communication techniques engage with our patients where they are already highly engaged—on their devices. Furthermore, the COVID‐19 pandemic has only expedited the expansion of new technologies in cardiovascular care, primarily telemedicine, that may facilitate many of these more personal conversations.^58^ However, the correct balance between digital and human contact should be further studied, elucidating the risk of digital fatigue versus the power of human presence.

## 3 FUTURE DIRECTIONS

A number of efforts will be needed to further advance this patient motivation paradigm in the coming years. Physicians and nurses providing preventive cardiology care will need to be trained to expand their knowledge of motivational interviewing techniques, the psychology of disease prevention, and to effectively clarify their patients' priorities, values and motivations. This will inform the most tailored, meaningful conversations. Training will also be needed to successfully approach these in the most professional, respectful, and effective manner. Additional time will have to be allotted to each visit so that these conversations can be appropriately accommodated, and clinicians will have to pay special attention to minimizing any commercial biases during these discussions. Dedicated time could be set aside during an initial patient visit to elicit these patient motivations, providing it almost as much emphasis as the social history or physical exam.

Formal research will be needed to measure the effectiveness of these approaches, although this may markedly vary across patients, in changing patient motivation, lifestyle habits, medication adherence, and risk factor control. Eventually the impact of this approach on clinical outcomes should be studied, although methodological challenges would include the need for very large samples sizes and long follow up, which may limit feasibility. Validated methodologies to measure patient satisfaction and motivation after these conversations should be developed and used. Tools that evaluate motivation toward lifestyle change and adherence to cardiovascular medications will have to be developed and used at the early stages of the preventive cardiology care process, so that potential droppers during follow‐up can be detected and communication efforts enhanced before this happens.

Research will need to be conducted to identify windows of opportunity for such motivational interventions. For the purposes of primary prevention, this may be following a cardiac event in the life of a close friend or family member. For secondary prevention, this window may open immediately following an ACS event or hospitalization. Although these times may be particularly effective, we pose that assessing and tackling patient motivations should become a natural component of preventive cardiology visits. Furthermore, the CVD field could adopt effective and innovative strategies from other fields. The diabetes field is particularly dedicated to understanding motivation of insulin self‐management, or lack thereof, using person‐reported outcome measures to focus encounters on life domains important to the patient.^59^

## 4 CONCLUSIONS

CVD remains the number one killer in the US. This continues despite identification of key lifestyle preventive interventions and availability of a myriad of effective pharmaceuticals. Education and intervention are the primary tools of modern preventive cardiology, and awareness surrounding CVD and its risk factors among the lay community has never been greater. Education, as the pillar of CVD prevention, has been shown to translate into motivational impact.^60^ However, long‐term adherence to lifestyle changes and medications remain key unmet needs. To enhance outcomes, we can now go further and address one of the key remaining barriers to cardiovascular health: motivation. The science of CVD prevention is relatively well understood; it is the *art* of prevention that now may require more investment. Doing so will require a paradigm shift in the approach to the clinician‐patient relationship, with more consult and follow‐up time dedicated to the emotional, psychological, and socioeconomic mindset that patients bring to the decisions they make about their health. This is currently foreign territory for our field and will require specific training and ability to engage in challenging conversations with patients. Although further research is needed, this paradigm change will most likely result in a deeper bond with our patients, improve their satisfaction with their care, and most importantly, improve their health outcomes.

## CONFLICTS OF INTEREST

The authors declare that they have no conflicts of interest relevant to the content of this manuscript.
